# The effects of decentralisation on patient and service outcomes: a case of the 2018 decentralisation of multidrug-resistant tuberculosis in Zambia

**DOI:** 10.1186/s13690-025-01672-7

**Published:** 2025-07-24

**Authors:** Tulani Francis L. Matenga, Malizgani Paul Chavula, Joseph Mumba Zulu, Adam Silumbwe, Patricia Maritim, Margarate N. Munakampe, Batuli Habib, Namakando Liusha, Jeremiah Banda, Ntazana N. Sinyangwe, Chris Mweemba, Angel Mubanga, Patrick Kaonga, Mwiche Musukuma, Henry Phiri, Hikabasa Halwiindi

**Affiliations:** 1 Department of Health Promotion, School of Public Health, University of Zambia, Lusaka, Zambia; 2 Department of Community and Family Medicine, School of Public Health, University of Zambia, Lusaka, Zambia; 3 Department of Epidemiology and Global Health, Umeå University, 901 87 Umeå, Sweden; 4 Department of Health Policy and Management, School of Public Health, University of Zambia, Lusaka, Zambia; 5 Yakini Health Research Institute, Lusaka, Zambia; 6 Ministry of Health, Kitwe Teaching Hospital, Off Kumboka Drive, P.O. Box 20969, Kitwe, Zambia; 7 Department of Epidemiology and Biostatistics, School of Public Health, University of Zambia, Lusaka, Zambia; 8 Department of Environmental Health, School of Public Health, University of Zambia, Lusaka, Zambia; 9 Ministry of Health, Ndeke House, Haile Selassie Avenue, P.O. Box 30205, Lusaka, Zambia

**Keywords:** Decentralisation, Patient care, Health services, Multidrug-resistant tuberculosis, Zambia

## Abstract

**Introduction:**

The Zambian government decentralised tuberculosis control programs by transferring responsibility for the care and treatment of multidrug-resistant tuberculosis (MDR-TB) patients from a two-national hospital model to provincial hospitals and other lower-level healthcare structures. Limited evidence exists on the effects of decentralisation on the quality of TB care provided through public sector decentralisation. In this paper, we explored the impact of decentralising MDR-TB on patient and service outcomes.

**Methods:**

This study used a mixed-methods approach. Quantitative data were collected through a survey of 244 MDR-TB patients, while qualitative data was collected through interviews with TB coordinators, healthcare providers, patients, and caregivers. Participants were drawn from health facilities and the Ministry of Health. Quantitative data was analysed in STATA version 16.0, while thematic analysis was used for the qualitative data.

**Results:**

Decentralisation has improved patient care and management by increasing access to essential commodities such as medication and diagnostic testing. It has led to more equitable distribution of MDR-TB healthcare services and resources across different population groups, regardless of social, economic, or demographic factors. Furthermore, the quality of life for MDR-TB patients has improved, with better adherence to medication resulting from increased family support. Due to decentralisation, tailored community and patient-centred services have been integrated resulting in reduced congestion at facilities. The study also identified challenges, including heavy workload for healthcare staff, fragmented coordination of supervisory responsibilities, and confusion over roles in patient management, which negatively impacted the decentralisation process.

**Conclusion:**

The decentralisation of MDR TB services offers significant benefits but is not a guaranteed solution, as poor planning or implementation can lead to challenges in service delivery.

**Table:** 

**Text box 1.** **Contributions to the literature**	
• This study fills a critical gap by providing robust evidence on how the decentralisation of TB services in the public sector influences the quality of care, particularly in low-resource settings, where health system challenges are most pronounced. • By examining patient outcomes within decentralised TB care models, the study highlights the direct impact of service delivery reforms on population health, including treatment adherence, equitable access, and disease control. • The findings offer actionable insights for policymakers and health practitioners, emphasising the need for tailored, context-specific strategies. This approach can empower them to optimise decentralisation benefits while mitigating potential risks, ultimately advancing effective TB control and improving health equity at the community level.	

## Introduction

In 2023, an estimated 10.8 million people were affected by TB, with 8.2 million new cases reported—the highest ever recorded [32]. The global TB incidence was 134 per 100,000 population, with 662,000 (6.1%) of new cases co-infected with HIV and 400,000 (3.7%) being multidrug-resistant (MDR) or rifampicin-resistant (RR) TB. MDR/RR was 3.2% among new cases and 16% among previously treated cases. TB remained the leading cause of death from a single infectious disease globally, with the number of fatalities nearly twice the number of HIV/AIDS-associated deaths [7]. Regionally, TB incidence varied, with the majority of cases in Southeast Asia (45%), Africa (24%) and the Western Pacific (17%). Five Asian countries accounted for 56% of the global TB cases [11].

TB remains a significant public health challenge in Zambia, accompanied by a high HIV/AIDS prevalence and widespread poverty [9]. In 2022, the country was among the 30 highest-burden countries for TB/HIV and MDR-TB, with an estimated burden of TB of 69,000 cases and 1,900 MDR-TB cases. Like many low- and middle-income countries (LMICs), Zambia faces significant challenges in detecting and managing MDR-TB, leading to poor treatment outcomes over the years [31].

The World Health Organisation (WHO) recommends that patients with MDR-TB be treated primarily through ambulatory care [30] provided by grassroots or community workers, either in peripheral facilities close to or at the patient's residence for the majority of the treatment period [33]. This type of provision of care is known as the decentralisation of MDR-TB services. Health systems in many African countries have undergone significant reforms, with decentralisation of health services being central to these changes [4]. The intention is to promote accountability and local preference and to make health systems more equitable, inclusive, and fair [25]. In 2018, the Zambian government decentralised tuberculosis control programs by transferring responsibility for the care and treatment of MDR-TB patients from two national hospitals to provincial hospitals and lower levels of the health system [20].

Decentralisation is bound to have varying levels of success in achieving its intended effects on population health outcomes and health services. The benefits of decentralisation include cost savings for MDR-TB patients [15], reduced loss to follow-up [22], improved case detection [8], higher patient success rates [14, 16] and improved quality of healthcare. On the other hand, challenges of decentralisation include inadequate funding, delayed treatment, difficulties linking from testing to care, inadequate infrastructure, and lack of support from stakeholders [17].

Despite the perceived success of MDR-TB decentralisation, it’s argued that the quality of public sector MDR-TB care is scattered and anecdotal, and whether it improves patient and service outcomes remains a topic of curiosity [14, 24]. It is against this background that this paper aims to explore the effects of decentralising MDR-TB on both patient and service outcomes. Health sector stakeholders need to understand these effects and develop strategies that maximise the benefits and mitigate the challenges of decentralisation reforms across various settings [1].

## Methods

### Study design

This study was part of a large research project to document and evaluate the impact of decentralising the programmatic management of MDR-TB in Zambia using a mixed-methods approach [6, 34]. The study employed a concurrent triangulation, mixed-methods, cross-sectional study design. In this paper, we focus on the effects of decentralising MDR-TB on patient and service outcomes.

### Study setting and context

To explore the effects of decentralising MDR-TB on both patient and service outcomes, data were collected from a random sample of 30 MDR-TB treatment sites in 30 districts across all 10 provinces. The study included a variety of healthcare facilities, including both provincial and district hospitals. The 30 sites out of the total 100 MDR-TB treatment sites were randomly selected for inclusion in the study; see [6] for a detailed description of the methods section.

### Study population and sampling

The study examined the extent to which the decentralisation policy had led to an improvement in the quality of services provided for MDR-TB at decentralised sites.

#### Quantitative component

A sampling framework comprising 117 MDR-TB treatment sites in the country was used to randomly select the 30 health facilities. All patients at the health centres chosen were eligible to participate in the interviews. The study specifically enrolled MDR patients who were of consenting age (adults aged 18 years +) with a laboratory-confirmed diagnosis of MDR-TB. All patients attending the MDR-TB clinic were enrolled in the study, and those who provided consent for participation were included. The quantitative component of the survey collected data from individuals who were MDR-TB patients and focal point persons working in the TB unit from various sites. A sampling framework was employed that encompassed all 117 MDR-TB treatment facilities nationwide. From this list, 30 health facilities were randomly chosen. In total, 244 MDR-TB patients were sampled: 64 (26.2%) were female and 180 (73.8%) were male.

Additionally, 24 healthcare facility providers, one from each facility, were selected based on their expertise in managing MDR-TB. These providers completed a questionnaire assessing various aspects of MDR-TB services following decentralisation. The questionnaire covered topics such as the quality of services provided, improvements in service delivery, and other related issues.

#### Qualitative component

We purposively sampled 10 health treatment centres from 10 districts in five provinces in Zambia. The selection took into consideration location, patient volume, performance, and other unique contextual characteristics that would help in developing an overarching understanding of how the decentralisation process occurred. Due to the homogeneous nature of the targeted participants, 5–10 interviews were conducted at each health facility with different cadres. Individuals were purposively sampled for either playing a key role in delivering MDR-TB services or being recipients of MDR-TB services at the facility. Key informants included TB coordinators at national, provincial, district, and facility levels, as well as in-depth interviews with healthcare providers at both health facility and community levels. Additionally, interviews were conducted with MDR-TB patients and their caregivers.

### Data collection methods and analysis

#### Quantitative component

Data was collected through questionnaires administered by trained data collectors. Two different data collection tools were used: a health facility questionnaire and a patient questionnaire. The patient tool was administered to MDR-TB patients. The health facility tool was administered to health facility workers who were knowledgeable about managing MDR-TB services at their respective facilities.

Aggregated data were collected using the online platform Kobo Toolbox and analysed using Stata 16 software (StataCorp, College Station, Texas, USA). Descriptive statistics were used to analyse participant characteristics, including demographic and clinical characteristics. Data was summarised into mean and median distributions at both facility and aggregate levels.

Table 1 shows the distribution of the 244 MDR-TB participants. Of those interviewed, 26.2% (*n* = 64) were female participants, and 73.8% (*n* = 180) were male participants. The highest number of MDR-TB patients were from the Copperbelt (27.5%, *n* = 67), Lusaka (27.5%, *n* = 67), and Southern (10.1%, *n* = 25) provinces, while the least number of participants were from the North-western (2.1%, *n* = 5) and Eastern (3.3%, *n* = 8) provinces.

**Table 1:** Distribution of patients with multidrug-resistant tuberculosis interviewed by gender and province in Zambia in the study period, 2023

Province	Female *n* = 64	Male *n* = 180	Total *N* = 244
Central	3 (4.7)	20 (11.1)	23 (9.4)
Copperbelt	19 (29.7)	48 (26.7)	67 (27.5)
Eastern	0 (0.0)	8 (4.4)	8 (3.3)
Lusaka	13 (20.3)	54 (30.0)	67 (27.5)
Muchinga	5 (7.8)	10 (5.6)	15 (6.2)
North-western	1 (1.6)	4 (2.2)	5 (2.1)
Northern	6 (9.4)	16 (8.9)	22 (9.0)
Southern	9 (14.1)	16 (8.9)	25 (10.1)
Western	8 (12.5)	4 (2.2)	12 (4.9)

#### Qualitative component

A total of 112 interviews were conducted to generate information on the decentralisation of MDR-TB services. Key informant interviews provided valuable insights from coordinators at various levels of the health system, offering unique perspectives on implementation and policy. In-depth interviews with community-based providers explored their roles and experiences in delivering MDR-TB services. Patient and carer interviews were conducted to gain a deeper understanding of their experiences, challenges, and perspectives throughout the treatment process. By including participants from diverse backgrounds and regions, the study aimed to obtain a comprehensive understanding of the decentralisation of MDR-TB services in Zambia and the factors influencing its implementation. Table 2 summarises the qualitative interviews conducted with respondents in each category during the data collection period.

**Table 2:** Categories of participants interviewed on decentralisation of multidrug-resistant tuberculosis services in Zambia, in the study period, 2023

Interview Category	Total	Health System Level Distribution	Health System Levels Represented per City
**Nurses**	18	Facility: 12 District: 3 Provincial: 3	Facility, District, Provincial (each city) National (Lusaka, as relevant)
**Community Health Workers**	17	Facility: 12 District: 3 Provincial: 2	Facility, District, Provincial (each city) National (Lusaka, as relevant)
**Patients**	32	Facility: 32	Facility (all patients)
**Caregivers**	21	Facility: 21	Facility (all caregivers)
**Key Informants (Managers)**	24	Facility: 7 District: 7 Provincial: 7 National: 3	Facility, District, Provincial (each city) National (Lusaka, as relevant)
**Total**	**112**		

### Data analysis (qualitative)

All interviews were audio-recorded and transcribed verbatim. Transcripts were read and re-read to allow for familiarisation and to start the process of open coding. Coding was performed inductively, without following predetermined codes. Codes were then grouped into clusters around similar and interrelated ideas, from which several themes emerged. The development of themes was guided by the results obtained from the quantitative data, aligning with the study objectives [13]. TFLM, PM, MM, MPC, and BPH performed preliminary data analysis for the main study. For this paper, a further analysis focusing on the effects of decentralisation on patient and service outcomes was conducted by the first author (TFLM). A narrative summary with illustrative quotes was then used to present the case descriptions and themes from the interviews, along with their corresponding assertions.

## Results

This section examines the impact of decentralising MDR-TB on patient and service outcomes. It delves into the various aspects, including improvements in pharmacy supplies, patient outcomes, availability of medical equipment and laboratory services, and community involvement. Furthermore, it examines the impact of decentralisation on patient adherence to medication, patient-centred services, and the overall quality of health while also acknowledging the challenges encountered during this transition. The insights presented here are derived from healthcare providers and qualitative interviews with key informants, providing a nuanced understanding of the decentralisation experience.

### Positive effects of decentralisation of multidrug-resistant tuberculosis

A total of 24 healthcare providers were asked various questions regarding MDR-TB service provision after decentralisation, including the quality of the services offered, improvements in these services, and others. Responses were rated as agree, neutral, and disagree. The table below shows their responses. Table 3 below presents the views of staff regarding MDR-TB decentralisation.

**Table 3:** Study views of Tuberculosis clinic staff regarding service improvements following the 2018 decentralisation of multidrug-resistant tuberculosis services in Zambia

Staff response *N* = 24 (%)			
Question/statement	Agree	Neutral	Disagree
Before decentralisation, there were inadequate pharmacy supplies	15 (62.5)	4 (16.67)	5 (20.83)
Decentralisation led to better patient outcomes	23 (95.83)	0 (0.00)	1 (4.17)
Decentralisation led to many changes for better TB services	24 (100)	0 (0.00)	0 (0.00)
Decentralisation led to confusion over supervisory responsibility	2 (8.33)	3 (12.50)	19 (79.17)
Decentralised MDR-TB policy led to community empowerment and ownership	14 (58.33)	3 (12.5)	7 (29.17)
Decentralisation led to more medical equipment	14 (58.33)	4 (16.67)	6 (25.00)
Decentralisation led to more laboratory services making appropriate diagnoses	12 (50.00)	5 (20.83)	6 (25.00)
Decentralisation resulted in the facility being more equipped to support HIV treatment	16 (66.67)	5 (20.83)	3 (12.50)
At this facility, decentralisation has led to the efficient integration of TB and HIV services	19 (79.17)	3 (12.50)	2 (8.33)

#### Improved pharmacy supplies

The study found that the majority of the healthcare providers (62.5%) agreed that pharmacy supplies were inadequate before decentralisation, indicating that decentralisation led to an improvement in the availability of pharmacy supplies.

Qualitative findings revealed that the pharmacy supply chain mechanism was strengthened by the presence of dedicated MDR-TB pharmacy focal persons at most large facilities. Additionally, provincial hubs communicated directly with the National Tuberculosis and Leprosy Program pharmacists to address logistical needs. This enhanced supply chain mechanism leading to improvement in the availability of MDR-TB medication at local facilities and reduced dependency on central facilities.

*We have allowed other facilities to have enough stock of the drugs for the patients so that they can monitor the client as the client waits for that big review at a central hospital*.” [Interview with TB coordinator, District Office].

#### Workload due to supervisory roles

The study explored the perceptions of healthcare providers on whether decentralisation had led to confusion over supervisory responsibilities. Among the 24 facilities that responded to this question, 79.17% (19/24) disagreed that decentralisation led to confusion over supervisory responsibility. Only 8.33% (2/24) of staff members agreed that decentralisation led to confusion over supervisory roles, while 12.5% (3/24) remained neutral.

Findings also revealed that in some cases, the decentralisation process had resulted in additional workload required to manage MDR-TB patients. This created mixed reactions, with some healthcare workers being more motivated to work because they had received technical and financial support to manage MDR-TB. In contrast, others complained about the heavy workload due to additional roles and responsibilities.

*Another thing I can mention is human resources; you find that at a facility, there are only two people, so one is working from MCH, and another one is working somewhere, so you cannot even add another program because they are limping. Therefore, human resources and the same human resources should be trained to implement the existing activity*. [Interview with District TB coordinator].

#### Access to essential commodities

The impact of decentralisation on the availability of medical equipment was examined. About half, 58.33% (14/24) of the facilities reported that decentralisation led to having more medical equipment, while 25% (6/24) thought otherwise. Furthermore, decentralisation was perceived to have improved the ability of laboratory services to make accurate diagnoses, with 50% (12/24) of health workers affirming this. In comparison, 25% (6/24) disagreed that decentralisation led to more medical equipment.

Qualitative findings confirmed that as healthcare services were moved closer to communities through decentralisation efforts, the consistent availability of essential commodities, such as diagnostic and testing equipment**,** was also observed.

*It has helped us because of the availability of Genexpert machines. It has enabled us to diagnose cases much faster than sending samples to Lusaka*. [Interview with TB Coordinator Provincial office].

#### Community involvement and ownership

The study investigated whether decentralisation of MDR-TB led to increased community involvement, participation in decision-making, and a sense of ownership over local TB programs. Of the 24 facilities surveyed, 58.33% (14/24) reported that the MDR-TB had fostered these aspects of community empowerment and ownership. However, 8.33% (2/24) disagreed, and 20.83% (5/24) strongly disagreed with this assessment.

In the qualitative findings, engaging the community at various levels, including local leadership, was reported to have significantly contributed to raising awareness about the services. This involvement helped ensure that the community was well-informed and fostered a sense of ownership over the program, which in turn enhanced local efforts to promote and sustain awareness.

*The process of engaging the community to recognise that several factors can give us TB. You can be on a bus, and a person is sitting next to you who is coughing; they do not have the etiquette to cover their mouth, and you can get it. This one is even curable, so through our ongoing engagement with the community and local leadership, we aim to ensure that this time, it's not restricted to just healthcare personnel*. [Interview with district TB manager].

#### Improved adherence to medication

The treatment outcomes of the MDR-TB patients were investigated to determine the proportion of those who had ‘cured’, ‘completed’, ‘died’, and ‘failed’ outcomes. Table 4 below presents treatment outcome indicators before and after decentralisation, which were used as proxy measures for medication adherence among MDR = TB patients. In this analysis, medication adherence was not assessed using direct outcome measures, such as the number of missed doses or appointments. Instead, outcome measures— including the proportion of patients cured, those who completed treatment, and those who experienced treatment failure or death—were used to reflect overall adherence. Higher rates of cure and treatment completion and lower rates of failure and loss to follow-up are generally indicative of better adherence to prescribed medication regimens. These outcome-based parameters provide an indirect assessment of adherence by capturing the result of patient's engagement with their treatment. The facility survey also examined specific treatment outcomes of interest in all MDR-TB facilities to gauge the effect of decentralisation; this information is presented in Table 4, which shows overall outcomes for all health facilities. There was a total of six outcomes that were being tracked, and these included the total number of MDR-TB cases cured, the total number of those that had completed treatment, the total number of deaths from MDR-TB, the total number of failed treatments, lost to follow up and the numbers that were not evaluated. The aim was to examine whether there was an improvement in these treatment outcomes before and after the decentralisation.

**Table 4:** Patient outcomes in the decentralisation of multidrug-resistant tuberculosis services in Zambia, during the study period, 2018–2023

Indicator	Before N (%)	Total patients	After N (%)	Total patients
Cured	39 (31.20)	125	148 (31.02	477
Completed	54 (43.20)		160 (33.54)	
Died	20 (16.00)		73 (15.30)	
Failed	4 (3.20)		30 (6.29)	

The proportion of cured cases showed a slight decrease from 31.20% after decentralisation to 31.02% after decentralisation. The proportion of patients who completed treatment declined from 43.20% to 33.54%. Although the absolute number of MDR-TB increased, the death rate remained relatively stable due to an overall increase in the total number of patients. Treatment failure rates rose from 3.20% to 6.29%, which may reflect improved tracking and reporting following decentralisation. Additionally, the proportion of patients lost to follow-up increased from 0.4.80% to 6.50%.

Interviews with key informants and healthcare providers revealed that the decentralisation of MDR-TB played a significant role in promoting patient adherence to treatment. This was due to local systems that supported patients in treatment and ensured adherence. These local systems included treatment supporters identified by the community, who followed patients and, in some cases, delivered medication to them in their communities.

*The adherence is better now. As you may be aware, we have treatment supporters, even for ourselves, when we notice that a patient has not been coming. Due to a lighter workload, we can make that phone call or follow up using the treatment supporters, so I think adherence has improved*. [Interview with key informant, Provincial health office].

#### Positive effects on patient outcomes

MDR-TB deaths are also an important indicator of program performance, and these deaths were tracked both before and after the decentralisation**.** A total of 20 (16. %) deaths were recorded in the period before decentralisation, while 73 (15.30%) deaths were recorded after decentralisation out of the total 477 patients recorded in the facilities. This shows a slight reduction in the number of MDR-TB patients.

Qualitative findings showed that with the coming of decentralisation, there has been a fair and just distribution of MDR-TB healthcare services, resources, and opportunities across different population groups, regardless of social, economic, or demographic factors. This has led to a reduction in the number of MDR-TB patients, which contributed to earlier identification and improved management of cases, potentially reducing the number of advanced or undiagnosed cases over time.

*It was good because you could see the needs of that person at home, how they stay and what they are eating and also the development in treatment on how they are responding because they did not have to walk long distances; instead, we used to follow them and able to see the improvement, but if we know that they have something else we send them at the facility to get checked*. [Interview with Community Health Worker-Health facility].

#### Patient-centred services

Decentralisation of MDR-TB has allowed healthcare services to be tailored to the specific needs of local communities, such as community follow-ups by community health workers (CHWs). The efforts of community health workers include providing support and advocating for resources to help patients navigate the decentralised system, including assistance with transportation, translation services, psychosocial support, and patient monitoring.

*Then, those who are already patients sometimes go into the communities for contact tracing; we call it contact tracing. For example, for a patient who is on TB medication, we speak with that patient. Can we come to your place because TB in the sputum is infectious? We follow them in the community to do contact tracing, and we also go into the wards to do contact tracing, especially for the bed-siders*. [Interview with TB Supporter, health facility].

Patients noted that they were treated with kindness and respect at the facilities and by CHWs within the community. Healthcare providers were perceived as encouraging and supportive, providing counselling to promote adherence to treatment regimens. They reinforced the importance of staying on track by sharing the experiences of patients who had previously failed to adhere, emphasising the consequences of non-compliance and the positive outcomes of persistence. Moreover, the providers were situated within the community, which increased patients'trust in the services. Regular follow-up through community-based structures also provided a supportive system for the patients.

*A decentralised system is the best. It's the best because patients now have an interface with us; they know us because we are all locals in the province. So, you can even give them your number, and they can call but I can imagine that time when it was in Lusaka* [KII Provincial Coordinator Western Province].

#### Reduced congestion at facilities

Decentralisation was reported to have decreased congestion at facilities because patients had more options for accessing services, reducing the time spent waiting to be attended to. Furthermore, because patients could quickly access health facilities, the number of patients who stayed at the health centre decreased. Due to the reduced congestion, patients and healthcare workers were also able to interact with patients more effectively.

*Additionally, at least the patients go home early, and they have plenty of rest and time to recuperate. We can talk to patients on a one-on-one basis because if there are many, you will be rushing to finish, but if there are few, at least one patient can open up to you if they have an inner problem.* [Interview with TB Supporter, Lusaka District].

#### Improved quality of health

In the qualitative interviews, patients reported that with the introduction of the decentralisation of MDR-TB services in most parts of Zambia, there was an improvement in the quality of health for MDR-TB patients, resolution of symptoms, and improved general condition, as patients were able to follow treatment regimen without missing doses and review dates. For example, one patient was initially unable to walk but later regained the ability to walk.

*From the time I was diagnosed, I think I am much better, and I am taking my treatment well. I am also able to work just as much as I used to before I was diagnosed with TB, and after I was found to have TB, I couldn't walk longer distances, and I couldn't even work; I am now better* (IDI, Female, Patient, Solwezi).

*He looks healthier now compared to the way he was in the past, and I mentioned it to say that when he got sick, it was very severe, to the point where he couldn't talk, sit on his own, or do anything* (IDI, Caregiver, Kitwe).

Prompt diagnosis and initiation of appropriate treatment are crucial for preventing the further transmission of the disease, improving patient outcomes, and reducing the risk of more severe health outcomes. This improvement has been observed by healthcare providers in the study due to the timely diagnosis and treatment of MDR-TB following the decentralisation of MDR-TB services.

*Earlier diagnosis is beneficial because the sooner the diagnosis is made, the better the outcome for both the patient and the program. Additionally, apart from benefiting the patient, it also reduces the risk of transmission, as patients are not delayed in diagnosis, which means fewer become exposed to the index case, and the problem will not grow. Therefore, it highlights the importance of infection control in the community, as well as the need to improve patient outcomes and reduce the burden on the institution.* [Interview with a key informant, district health office].

### Challenges associated with decentralization of multidrug-resistant tuberculosis

Decentralisation of MDR-TB management has the potential to improve access to care and patient outcomes. Still, it's essential to acknowledge the associated challenges that come with it to ensure its effectiveness and sustainability. Some of these challenges include a lack of necessary financial, human, and technical resources to manage and deliver healthcare services effectively, which may potentially compromise service quality and patient safety.

#### Staff attrition

Staff attrition significantly affects the decentralisation of MDR-TB treatment. Interviewees reported that staff leaving after undergoing training is a significant gap in the provision of services to patients. When trained staff leave, valuable knowledge and skills specific to MDR-TB management are lost, impacting the quality of care provided to patients.

*Several doctors were trained, but they left, making decentralisation a massive challenge. So, we had to struggle at some point, our partners would send people from Lusaka to come and do mentorship. So, the challenges were related to staff attrition; you train the staff today, and tomorrow, the number is halved, sometimes even less than that.* [Interview with key informant from Provincial Health office].

#### Inadequate Training

MDR-TB patients require healthcare workers to acquire new skills and knowledge to manage their conditions effectively. Participants reported a lack of access to ongoing professional development opportunities, which resulted in staff feeling ill-equipped and demotivated to provide services.

*The other barrier that we have at the moment is because of the new staff that has come who is still not yet acquainted with the system. That is one of the most significant barriers. As I mentioned, with this training not being aligned with these orientations…… that is the number one challenge.* **[**Interview with health care provider, general hospital].

#### Limited infrastructure

The qualitative findings highlighted significant challenges faced by some decentralised facilities in providing adequate care for MDR-TB patients due to a lack of essential infrastructure. For instance, several facilities were reported to be missing critical components such as designated spaces, specialised laboratories, and isolation rooms, which are vital for the proper management and treatment of MDR-TB. One TB Coordinator emphasised this issue.

*We currently lack a dedicated chest clinic. Given this, how do you plan to establish MDR-TB services? Would this involve building a new clinic? A sizable chest clinic within the district hospital may be suitable. Considering the specialised nature of TB treatment, such a facility would be essential for providing effective MDR-TB care* [Interview with TB coordinator].

## Discussion

This paper examined the impact of decentralising MDR-TB services on patient outcomes by gathering perspectives from clients, healthcare workers, and stakeholders. Our findings demonstrate that decentralisation significantly enhances service delivery by improving the quality of care, increasing accessibility, promoting patient-centredness, and reducing facility congestion at central facilities. However, despite the benefits, decentralisation also poses challenges, including staff attrition, inadequate training and limited infrastructure.

Structure improvements resulting from the decentralisation of MDR-TB treatment align with prior studies in SSA, such as Molla et al. [21] and Nyirenda et al. [23], which highlight the public health benefits of bringing treatment closer to the community level. By establishing local treatment centres within communities, decentralisation addresses the barriers faced by patients managing chronic or severe conditions such as MDR-TB. Evidence from Malawi, for example, shows that decentralisation reduced hospital bed occupancy by 38% and shortened the average hospital stay, reflecting increased health system efficiency and timely care. Our study found that lowering facility congestion shortened patient wait times and enhanced service delivery, underscoring the need to prioritise local treatment access—particularly in resource-constrained settings.

Patients in decentralised settings benefited notably from receiving care close to their homes, allowing for stronger family support and improved adherence. This is consistent with Daru et al. [10], who documented sustained favourable treatment outcomes when patients are managed within communities. Moreover, involving community members in planning and monitoring ensures cultural appropriateness and community ownership, which are essential to the success of MDR-TB programs. Thus, engaging the community can ensure that the community is aware and fully involved in health-related activities. Community engagement can further support the delivery of MDR-TB services by enhancing the relevance and legitimacy of the services [34].

Patient-related outcomes in our study revealed critical health implications of decentralising MDR-TB care. Patient-centred approaches that tailor services to local needs have expanded access and improved treatment success rates, aligning with the Global End TB Strategy's goals of emphasising equitable access to diagnosis, treatment, and care. Key enablers include community follow-ups, Directly Observable Therapy (DOT), patient education and social support, all of which promote adherence and reduce treatment defaulting [8]. Technological innovations and continued efforts to enhance patient-centred care hold promise for further gains in MDR-TB control.

Despite having adequate staffing with trained personnel, staff attrition emerged as a significant barrier to the public health system. High turnover increased workload and caused mixed reactions among healthcare workers, a challenge echoed in other studies [12, 26]. Decentralisation can also introduce coordination difficulties, as noted by Bolton [3] and Abimbola et al., [2]. Nonetheless, our findings highlighted the critical role of decentralised diagnostic facilities and reliable medication supply chains, supported by dedicated pharmacy focal point persons, which are vital for maintaining treatment continuity [28, 29]. Centralised TB services previously resulted in poor accessibility, patient dissatisfaction and inequities due to transport challenges and limited diagnostic resources [18]. Our study supports evidence that decentralisation promotes fair and just distribution of MDR-TB services, improving equity across socioeconomic and demographic groups. Sachdeva and colleagues [27] found that expanding Xpert MTB/RIF significantly increased TB case notification, while MacPherson [19] documented equitable TB care delivery, irrespective of deprivation levels, which contributed to patient satisfaction.

From a public health perspective, decentralisation combined with patient-centred care should be prioritised in TB control strategies, particularly in resource-limited settings. By focusing on local needs and ensuring that patients receive comprehensive support throughout their treatment, health systems can achieve better outcomes, reduce transmission, and ultimately contribute to the global goal of ending TB. This approach not only enhances the effectiveness of TB programs but also promotes equity and access to care for all individuals, regardless of their location or socio-economic status [5].

### Limitations and strengths of the study

Although facilities were randomly selected, participation at the patient and provider levels was based on consent and availability, which may have introduced selection bias and affected the generalizability of the findings. Both quantitative and qualitative data relied on self-reported information, which may be subject to recall bias or social desirability bias, particularly regarding sensitive topics such as treatment adherence or service challenges. Despite these limitations, the study provides robust and contextually relevant evidence on the decentralisation of MDR-TB services in Zambia, offering valuable lessons for similar health system reforms in other low- and middle-income countries. The use of quantitative and qualitative approaches enabled a comprehensive understanding of the effects of decentralisation of MDR-TB care in Zambia. This triangulation enhanced the validity and depth of the findings. Additionally, the study sampled across all 10 provinces, ensuring broad geographic representation and capturing variations in healthcare delivery and patient experiences. The inclusion of multiple respondent categories (patients, healthcare workers, TB coordinators, caregivers) provided a holistic view of the impacts of decentralisation.

## Conclusion

This study aimed to explore the impact of decentralising MDR-TB services on both patient and service outcomes. The findings demonstrate that decentralisation has yielded notable improvements across several key areas. By bringing MDR-TB care closer to communities, decentralisation enhanced the capacity of the healthcare workforce, enabling more efficient and response service delivery. The integration of TB and HIV services was streamlined, fostering a more holistic approach to patient care and facilitating better management of co-infections.

Decentralisation also empowered communities by involving them more directly in the care processes, promoting greater engagement and ownership of health outcomes. Timely service delivery improved as patients experienced reduced travel distances to access care, leading to increased convenience and potentially better adherence to treatment. Additionally, the decentralised model strengthened patient management systems, resulting in improved tracking of sputum culture samples, more effective monitoring of patient appointments and an overall increase in the number of patients served. The decentralisation of MDR-TB services has had a positive and multifaceted impact, improving both service delivery and patient outcomes. These findings underscore the value of decentralisation as a strategy for enhancing access, efficiency and quality of care in MDR-TB management.

## Data Availability

Qualitative data transcripts are available upon request from the author via email.
